# ^99m^Technetium SPECT–guided functional liver–sparing radiotherapy planning for recurrent hepatocellular carcinoma: a dosimetric comparison

**DOI:** 10.1007/s12194-026-01114-1

**Published:** 2026-08-07

**Authors:** Takuya Taniguchi, Kazutaka Miki, Kosei Adachi, Shuto Nakaya, Kaede Kawagchi, Osamu Tanaka, Masayuki Matsuo

**Affiliations:** 1 Department of Radiation Oncology, Asahi University Hospital, 3-23 Hashimoto-cho, Gifu, Gifu 500-8523 Japan; 2 Department of Radiology, Gifu University School of Medicine, 1-1 Yanagido, Gifu, 501-1194 Japan

**Keywords:** Hepatocellular carcinoma, IMRT, Function-preserving radiation therapy, VMAT

## Abstract

The aim of this study was to establish an optimal treatment strategy for functional liver–sparing radiotherapy by incorporating technetium-99 m galactosyl human serum albumin (^99m^Tc-GSA) single-photon emission computed tomography (SPECT) into radiotherapy planning for recurrent hepatocellular carcinoma (rHCC). Three irradiation techniques—volumetric-modulated arc therapy (VMAT), intensity-modulated radiotherapy (IMRT), and three-dimensional conformal radiotherapy (3D-CRT)—were systematically compared. Six patients with rHCC who underwent stereotactic body radiotherapy (SBRT) were included. Eligible patients had previously received SBRT to a different hepatic segment and had undergone ^99m^Tc-GSA SPECT before the current course of radiotherapy. For treatment planning, planning computed tomography (CT) images were rigidly registered with ^99m^Tc-GSA SPECT images, and functional liver regions were manually contoured by radiation oncologists. Using identical functional liver contours, three treatment plans—VMAT, IMRT, and 3D-CRT—were independently generated by medical physicists. The prescribed dose was 40 Gy, delivered in four fractions. These treatment plans were retrospectively created for research purposes and were separate from actual clinical treatments. Under identical planning target volume dose-coverage conditions, the dose–volume histogram indices (V2–V40) of the functional liver were compared among the three techniques. VMAT consistently demonstrated the best preservation of functional liver regions, particularly in the low-dose range, followed by IMRT and 3D-CRT. These findings demonstrate the feasibility of ^99m^Tc-GSA SPECT-guided function-avoidance radiotherapy planning for the re-irradiation of rHCC and suggest that VMAT provides an optimal balance between target coverage and functional liver preservation.

## Introduction

Hepatocellular carcinoma (HCC) remains one of the most common malignancies worldwide and is associated with a high recurrence rate even after curative treatment, with more than half of the patients developing intrahepatic recurrence within five years [1, 2]. A substantial proportion of patients with recurrent hepatocellular carcinoma (rHCC) have limited therapeutic options because of impaired liver function or multifocal disease.

Among the available treatment modalities for rHCC, radiotherapy (RT) plays an increasingly important role as a noninvasive local therapy for patients who are ineligible for surgery or ablation [3–7]. Recent advances in high-precision RT techniques, such as intensity-modulated radiotherapy (IMRT), volumetric-modulated arc therapy (VMAT), and stereotactic body radiotherapy (SBRT), have enabled conformal dose delivery with improved tumor coverage and normal tissue sparing [8, 9]. According to the American Society for Radiation Oncology (ASTRO) guidelines for primary liver cancers, advanced techniques are recommended because of their favorable local control rates and low incidence of severe hepatic toxicity [10]. The HyTEC (High-dose per fraction, Hypofractionated Treatment Effects in the Clinic) initiative further established dose–volume constraints to minimize the risk of radiation-induced liver disease (RILD), emphasizing the need for precise dose distribution tailored to individual hepatic tolerance [11].

However, conventional normal tissue dose constraints assume that hepatic function is uniformly distributed throughout the liver, and treatment-planning computed tomography (CT) is primarily used to evaluate anatomical liver structures rather than regional functional heterogeneity [11]. Hepatic function is often heterogeneous in patients with HCC because of cirrhosis, fibrosis, or previous irradiation, leading to variable radiation tolerance across different liver segments [12]. This heterogeneity highlights the importance of incorporating functional imaging into RT planning to preserve well-functioning hepatic parenchyma and reduce the risk of RILD [13].

Single-photon emission computed tomography (SPECT) using technetium-99 m galactosyl human serum albumin (^99m^Tc-GSA) provides a quantitative assessment of regional hepatic function by reflecting asialoglycoprotein receptor activity in hepatocytes [14]. Several studies have demonstrated that ^99m^Tc-GSA SPECT–derived functional maps provide a useful basis for dose–function analysis in liver SBRT. These investigations consistently show that incorporating functional information into inverse planning enables selective sparing of highly functional liver regions without compromising target coverage. Collectively, this body of evidence indicates that SPECT-guided planning can support more personalized and function-preserving radiotherapy strategies for patients with liver tumors [15–19].

Building on previous evidence demonstrating the clinical feasibility of incorporating ^99m^Tc-GSA SPECT-derived functional information into liver radiotherapy planning, the aim of the present study was to identify an optimal planning strategy for functional liver sparing in patients with recurrent hepatocellular carcinoma. Specifically, three delivery techniques—VMAT, IMRT, and three-dimensional conformal radiotherapy (3D-CRT)—were compared to determine which approach achieved the most effective functional liver preservation while maintaining adequate target coverage. This comparative analysis provides important insights into the optimization of functional image-guided radiotherapy and advances individualized hepatoprotective treatment strategies for rHCC.

## Materials and methods

### Study design and patient selection

This retrospective treatment planning comparison study included six consecutive patients with rHCC who underwent SBRT as re-irradiation for newly developed intrahepatic recurrence following prior SBRT delivered to a different hepatic segment. The initial treatment for primary hepatic lesions varied among patients and included modalities other than SBRT; however, all patients underwent SBRT for recurrent tumors. As part of the pre-treatment evaluation, planning CT and technetium-99 m galactosyl human serum albumin ^99m^Tc-GSA SPECT were acquired to assess anatomical structures and regional liver function prior to re-irradiation. All treatment plans were retrospectively generated for research purposes only and were independent of actual clinical treatments.

Patients were included only if their clinical course met the following sequence:

1. Prior SBRT delivery for primary HCC. The dose of the prior SBRT was standardized across patients, with all cases receiving either 40 Gy in 4 fractions or 44 Gy in 4 fractions.
2. Acquisition of ^99m^Tc-GSA SPECT images for functional liver assessment before re-irradiation and
3. SBRT performed as re-irradiation for intrahepatic recurrence following prior SBRT delivered to a different hepatic segment.

The final cohort consisted of six consecutive patients who fulfilled all inclusion criteria. In Case 4, although both the initial and recurrent tumors were located within segment 7 (S7), the recurrent lesion was located away from the edge of the previously irradiated PTV and was therefore considered spatially distinct rather than a true in-field recurrence. Patient characteristics are summarized in Table 1.

**Table 1:** Clinical characteristics of the six patients with recurrent hepatocellular carcinoma

Case No	Irradiated regions	Recurrent regions	Anatomical liver volume (cm^3^)	Functional liver volume (cm^3^)
1	S2/3	S5	1101.0	924.3
2	S2, S5	S3	1118.5	1080.1
3	S1, 2, 6, 7	S3/4	1005.9	944.6
4	S1, 2, 3, 4, 6, 7	S7	1165.6	1007.2
5	S5	S7	1272.4	1255.0
6	S5/6	S4	783.7	743.1

Clinical characteristics of the six consecutive patients with recurrent hepatocellular carcinoma included in this study, summarizing the previously irradiated liver segments, locations of intrahepatic recurrence treated with stereotactic body radiotherapy, and the volumes of the anatomical liver and functional liver derived from ^99m^Tc-GSA SPECT imaging

### Imaging acquisition and image fusion

#### Planning CT acquisition

Planning computed tomography (CT) was performed to provide an anatomical reference for target delineation, organ-at-risk definition, image registration with functional imaging, and radiation dose calculation. CT imaging was performed using a 16-slice multidetector CT scanner (Alexion, Toshiba Medical Systems, Japan) under an end-expiratory breath-hold to minimize respiratory-induced liver motion and ensure reproducible anatomical positioning.

The scanning parameters were as follows: a tube voltage of 120 kVp, automatic tube current modulation (noise index, 9.0), gantry rotation time of 0.75 s, helical pitch of 1.0, field of view (FOV) of 500 mm, reconstructed pixel size of 0.98 mm, and slice thickness of 2.0 mm. Images were reconstructed using a standard abdominal reconstruction kernel.

The planning CT dataset served as the reference image for all subsequent analyses. Anatomical structures, including the whole liver and organs at risk (stomach, duodenum, spinal cord, intestine, right kidney, and left kidney), were manually contoured on axial CT images using MIM Maestro™ (version 7.4.3) by an experienced radiological technologist, in accordance with institutional contouring protocols.

#### ^99m^Tc-GSA SPECT acquisition

^99m^Tc-galactosyl human serum albumin ^99m^Tc-GSA scintigraphy was performed to evaluate regional hepatic function for functional liver–sparing radiotherapy planning.

After intravenous administration of ^99m^Tc-GSA (185 MBq), dynamic planar imaging was initiated immediately to confirm tracer uptake and distribution. Dynamic images were acquired in the anterior projection using a dual-head gamma camera (Discovery NM830; GE Healthcare) equipped with a low-energy, high-resolution sensitivity (LEHRS) collimator with a frame duration of 30 s for a total acquisition time of 20 min.

Subsequently, SPECT was performed to assess three-dimensional regional hepatic function. SPECT data were acquired in step-and-shoot mode over 360°, with 60 projections at 6° intervals and an acquisition time of 25 s per view. The energy window was centered at 140.5 keV with a ± 10% window, and a zoom factor of 1.33 was used. The data were acquired using a 128 × 128 matrix, resulting in a pixel size of 3.32 mm.

Because the SPECT system used in this study was not equipped with an integrated CT component, CT-based attenuation correction was not available. Therefore, image reconstruction was performed using an ordered-subsets expectation maximization (OSEM) algorithm without attenuation correction, and axial SPECT images were reconstructed with a slice thickness of 2 mm. The reconstructed SPECT images were rigidly registered to the planning CT images and used for regional hepatic function assessment in functional liver–sparing radiotherapy planning.

#### Image fusion and functional liver delineation

Image fusion was performed to spatially integrate anatomical and functional information for functional liver–sparing radiotherapy planning. The ^99m^Tc-GSA SPECT images were rigidly registered to the planning CT images using MIM Maestro™.

Registration was conducted by aligning the anatomical liver contours and overall hepatic morphology between the two image datasets. Registration accuracy was visually verified by a board-certified radiation oncologist.

The SPECT images were displayed using window and color map settings optimized by an experienced radiological technologist to enhance visualization of physiologically preserved hepatic parenchyma.

Final functional liver contours were manually delineated by a board-certified radiation oncologist with more than 20 years of clinical experience. The functional liver was defined based on ^99m^Tc-GSA SPECT as regions showing relatively preserved tracer uptake, while areas with decreased accumulation were considered functionally impaired and excluded. The same functional liver contours were consistently applied across all treatment plans (VMAT, IMRT, and 3D-CRT) to ensure methodological consistency.

### Treatment planning (VMAT, IMRT, 3D-CRT)

Radiotherapy treatment planning was performed using a commercial treatment planning system (Monaco version 5.11; Elekta AB, Stockholm, Sweden). The gross tumor volume (GTV) was delineated based on planning CT and diagnostic imaging. The clinical target volume (CTV) was generated by applying a uniform 5-mm margin to the GTV. The planning target volume (PTV) was defined by applying patient-specific three-dimensional margins to the CTV. Internal margins were determined based on respiratory motion assessed using inspiratory and expiratory planning CT images. In addition, a setup margin was incorporated to account for residual positioning uncertainties. Functional liver and whole-liver contours were not modified to exclude the GTV, and therefore partial overlap between these structures and the GTV was present. Daily image guidance was performed in accordance with standard SBRT. All treatment plans were created using a dose calculation grid size of 2 mm with a prescribed dose of 40 Gy delivered in four fractions. Three planning techniques were evaluated for each patient: VMAT, IMRT, and 3D-CRT.

For 3D-CRT and IMRT, multiple coplanar and non-coplanar beam arrangements were employed in accordance with established SBRT planning principles to achieve highly conformal dose distributions with steep dose gradients. Specifically, both the 3D-CRT and IMRT plans consisted of eight coplanar and four non-coplanar beams selected to ensure adequate target coverage while minimizing dose to surrounding organs at risk. VMAT plans were generated using two full arcs comprising one clockwise and one counterclockwise rotation. IMRT and VMAT differ in beam delivery and optimization strategy.

IMRT employs multiple fixed gantry angles with intensity-modulated beams optimized individually, whereas VMAT delivers radiation continuously during gantry rotation, allowing simultaneous optimization of gantry speed, dose rate, and multileaf collimator motion. These differences result in distinct dose distribution characteristics, particularly in the low-dose region within the liver.

Dose constraints for the target volumes and organs at risk were established with reference to the recommendations of the American Association of Physicists in Medicine Task Group 101 (AAPM TG-101). Because TG-101 does not explicitly define dose constraints for a four-fraction regimen, the constraints were conservatively adapted from the recommended limits for three- and five-fraction schedules, considering biological dose equivalence and re-irradiation setting. In particular, dose constraints for the whole liver were defined based on these guideline recommendations and were consistently satisfied in all treatment plans.

In the present study, cumulative dose summation of the initial and recurrent irradiation plans was not explicitly performed. The initial treatment was stereotactic body radiotherapy, in which a 40 – 44 Gy was delivered to a limited target volume with a steep dose fall-off to the surrounding liver parenchyma. As a result, regions corresponding to the previously irradiated 40 – 44 Gy dose area in the initial irradiation appeared as functional defects on ^99m^Tc-GSA SPECT, whereas liver function was preserved in the surrounding low-dose regions.

Since conventional dose–volume histograms (DVH)–based evaluation assumes homogeneous organ function, regions with preserved uptake on ^99m^Tc-GSA SPECT were regarded as functional normal liver tissue, even if they had been exposed to low doses during the initial treatment. Therefore, in this study, dose constraints were applied to the functional liver defined by ^99m^Tc-GSA SPECT imaging, rather than to cumulative physical dose distributions. This approach was intentionally adopted to prioritize functional liver preservation based on imaging-derived hepatic function.

Functional liver regions derived from technetium-99 m galactosyl human serum albumin ^99m^Tc-GSA SPECT imaging were incorporated as avoidance structures during the inverse planning of IMRT and VMAT. During inverse planning, volume-based optimization objectives were applied to the functional liver at doses of 10, 20, 30, and 40 Gy to minimize the irradiated functional liver volume. The details of these optimization objectives are summarized in Table 2. Identical target and organ-at-risk contours, including functional liver regions, were consistently applied across all three planning techniques to enable direct dosimetric comparisons. In addition to inverse planning optimization, beam arrangement was adjusted based on the spatial relationship between the planning target volume (PTV) and functional liver regions. While the Conventional IMRT and 3D-CRT plans employ eight standardized coplanar beams and four non-coplanar beams. Based on this configuration, functional image-guided planning was individually adjusted for each patient. Specifically, beam angles passing through highly functional liver regions were selectively avoided or removed, and beam arrangements were adapted to preferentially spare functional liver while maintaining target coverage and complying with the dose constraints summarized in Table 2.

**Table 2:** Dose constraints for target volumes and organs at risk

Structure	Dose constraint	Notes
GTV	Dmax ≤ 50 Gy	Maximum dose within GTV
PTV	D95 = 40 Gy	Prescription dose (40 Gy in 4 fractions)
	Dmax ≤ 50 Gy	Hot-spot limitation to ensure dose homogeneity
Functional liver	V10, V20, V30, and V40	Reduce the volume accordingly
	V2–V40 evaluated	Used for dose–function analysis
Whole liver	D700 cc ≤ 15 Gy	Adapted from TG-101
Stomach	Dmax ≤ 25 Gy	Conservatively adapted from TG-101
Duodenum	Dmax ≤ 25 Gy	
Small bowel	Dmax ≤ 25 Gy	
Spinal cord	Dmax ≤ 18 Gy	
Kidneys (right)	Mean dose ≤ 15 Gy	

Dose constraints and optimization objectives for target volumes and organs at risk used in treatment planning, determined with reference to AAPM Task Group 101 recommendations and conservatively adapted for the four-fraction regimen, with functional liver regions derived from ^99m^Tc-GSA SPECT incorporated as avoidance structures, with volume-based optimization objectives applied at multiple dose levels to reduce irradiation of functional liver tissue

For the planning target volume, dose constraints of D95 = 40 Gy and Dmax ≤ 50 Gy were applied for all techniques. By satisfying these clinically established constraints, adequate target dose conformity and homogeneity were consistently achieved, allowing a fair comparison of functional liver dose distributions among the techniques.

### Dosimetric analysis for liver function sparing

DVHs were generated for each optimized treatment plan using the treatment planning system. The DVHs were calculated for the PTV and functional liver.

Based on these DVHs, dose–volume parameters for the functional liver were extracted. Specifically, functional liver volumes receiving at least 2, 4, 6, 8, 10, 15, 20, 25, 30, 35, and 40 Gy (V2–V40) were calculated to enable a detailed assessment of functional liver dose sparing across different irradiation techniques.

The dose distributions within the functional liver and surrounding anatomical structures were visually assessed using the treatment planning system, and differences in the spatial dose distribution patterns among the irradiation techniques were visually compared.

### Statistical analysis

Statistical analyses were performed to compare the dosimetric parameters of the three radiotherapy planning techniques (3D-CRT, IMRT, and VMAT).

For each patient, DVH parameters for the functional liver (V2–V40) were extracted from the corresponding treatment plans. Due to the small sample size and paired nature of the data, nonparametric statistical methods were applied.

Pairwise comparisons between the treatment techniques were conducted using the Wilcoxon signed-rank test. All statistical analyses were performed using Microsoft Excel (educational version; Excel-based statistical software). All tests were two-sided, and a p-value of less than 0.05 was considered statistically significant.

Target coverage was evaluated using PTV D98, D2, and conformity index (CI). The conformity index was calculated according to the RTOG definition using the 40 Gy prescription isodose volume.

## Results

### Functional liver visualization and dose distribution

**Fig. 1:**
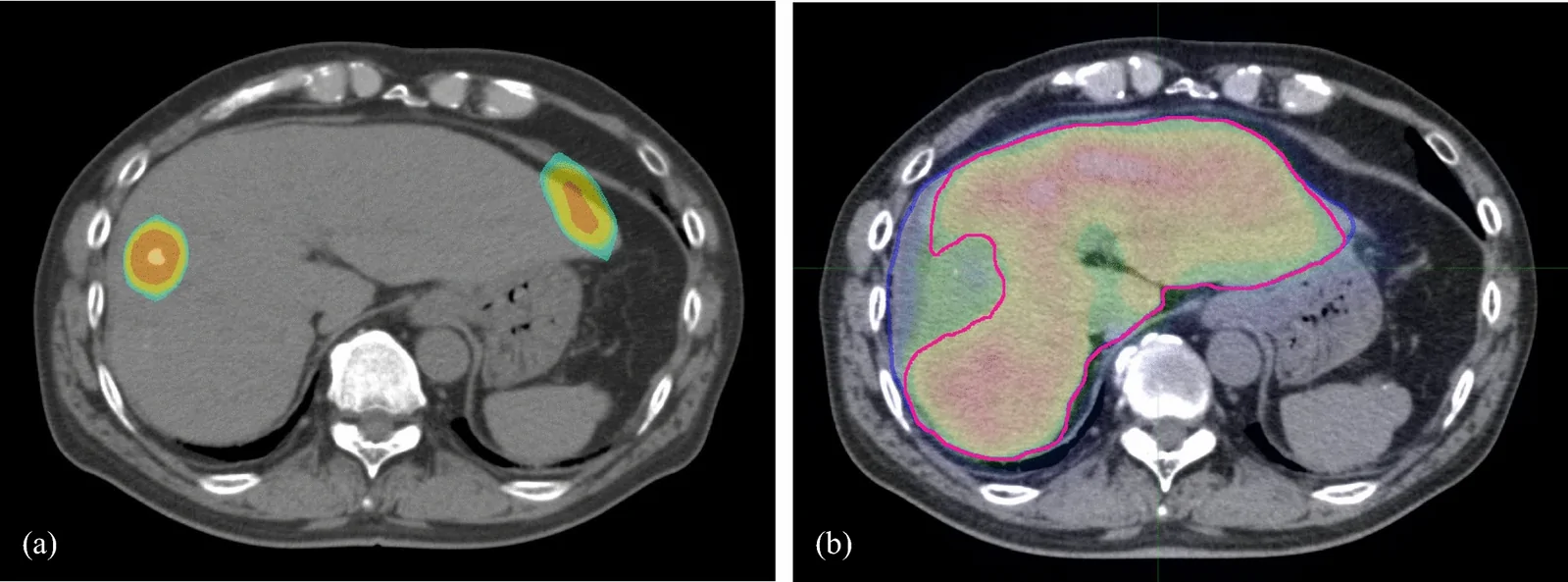
**a **Cumulative dose distribution of prior liver stereotactic body radiotherapy (SBRT) delivered to hepatic segments S2 and S5, illustrating the irradiated regions from the previous SBRT course. **b** Fusion of the planning computed tomography (CT) and ^99m^Tc-galactosyl human serum albumin (GSA) single-photon emission computed tomography (SPECT) images acquired approximately six months after the prior SBRT, with the anatomical liver delineated on the planning CT, and regional hepatic function visualized using a color map representing GSA uptake

Figure 1a illustrates the cumulative dose distribution of prior liver SBRT delivered to segments S2 and S5. This composite dose distribution represents the irradiated regions from the previous SBRT. Figure 1b shows the fusion of the planning CT and ^99m^Tc-GSA SPECT images used for functional liver visualization, acquired approximately six months after SBRT. The anatomical liver was delineated on planning CT, and regional hepatic function was visualized using a color map representing GSA uptake. Compared with the prior SBRT dose distribution shown in Fig. 1a, reduced GSA uptake was observed in previously irradiated liver segments, indicating decreased regional hepatic function. This spatial correspondence between regions corresponding to the previously irradiated 40–44 Gy dose area and reduced GSA uptake was visually confirmed by the treating radiation oncologists, consistent with previous reports demonstrating radiation-induced functional decline in the liver [20]. Functional liver contouring was performed based on these SPECT findings. This image fusion enabled the identification of spatial heterogeneity in liver function within the anatomy of the liver and provided a functional basis for subsequent treatment planning. Consistent with previous DVF-based analyses [17], regions corresponding to the prior 40–44 Gy irradiation area exhibited reduced ^99m^Tc-GSA uptake, indicating regional functional decline and contributing to spatially heterogeneous liver function.

**Fig. 2:**
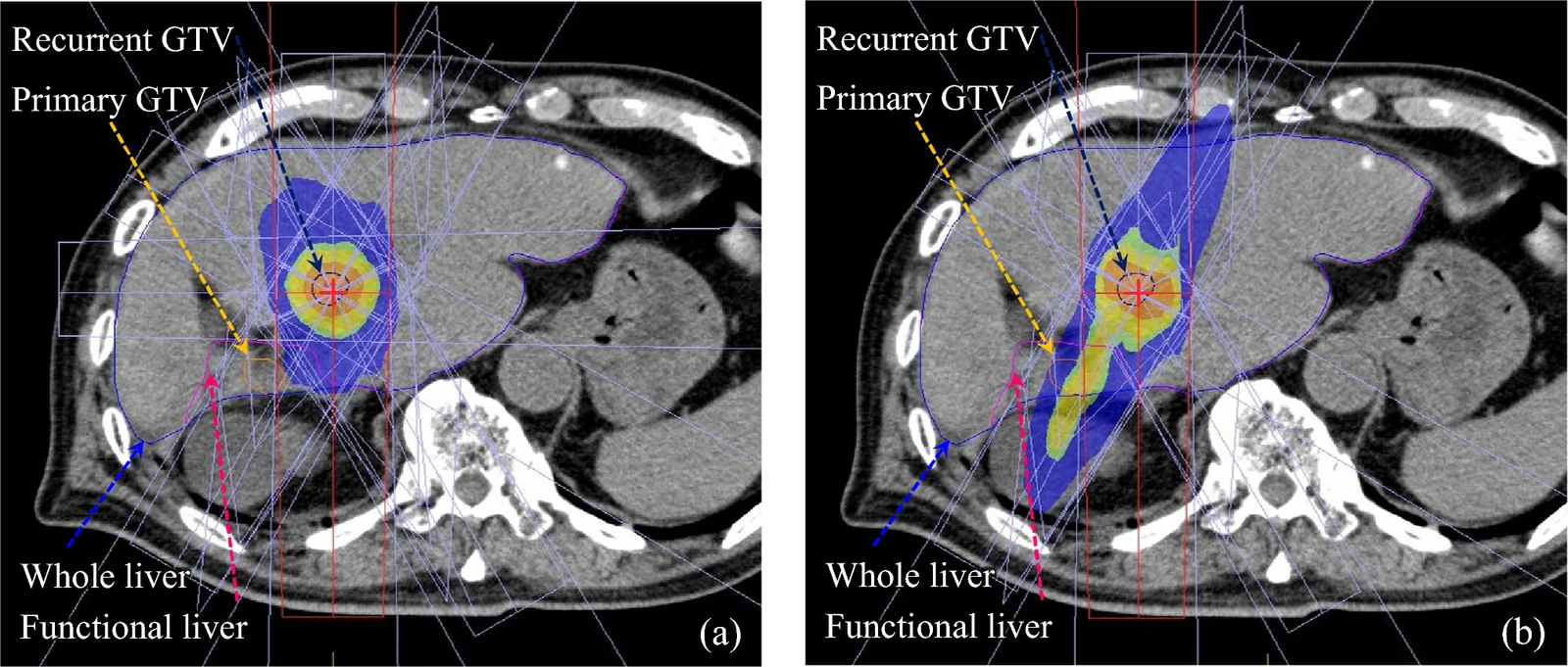
Comparison of beam arrangements and dose distributions between conventional and functional image–guided planning. **a** Conventional plan without functional liver information. **b** Functional image–guided plan incorporating ^99m^Tc-GSA SPECT. The primary lesion (previously irradiated region), recurrent target lesion, whole liver, and functional liver are shown. Although the primary lesion is located within the anatomical liver contour, it is excluded from the functional liver contour due to reduced ^99m^Tc-GSA uptake following prior SBRT. In the functional image–guided plan, beam arrangement weighting was applied to reduce irradiation to regions with preserved hepatic function

Figure 2 shows a comparison between conventional planning without functional liver guidance and functional image–guided planning incorporating 99mTc-GSA SPECT. The primary lesion (previously irradiated region), recurrent target lesion, whole liver, and functional liver are displayed to clarify their spatial relationships. Although the primary lesion is located within the anatomical liver contour, it is excluded from the functional liver contour due to reduced GSA uptake following prior SBRT. In the functional image–guided plan, beam arrangement and dose distribution were adjusted to reduce irradiation to regions with preserved hepatic function.

**Fig. 3:**
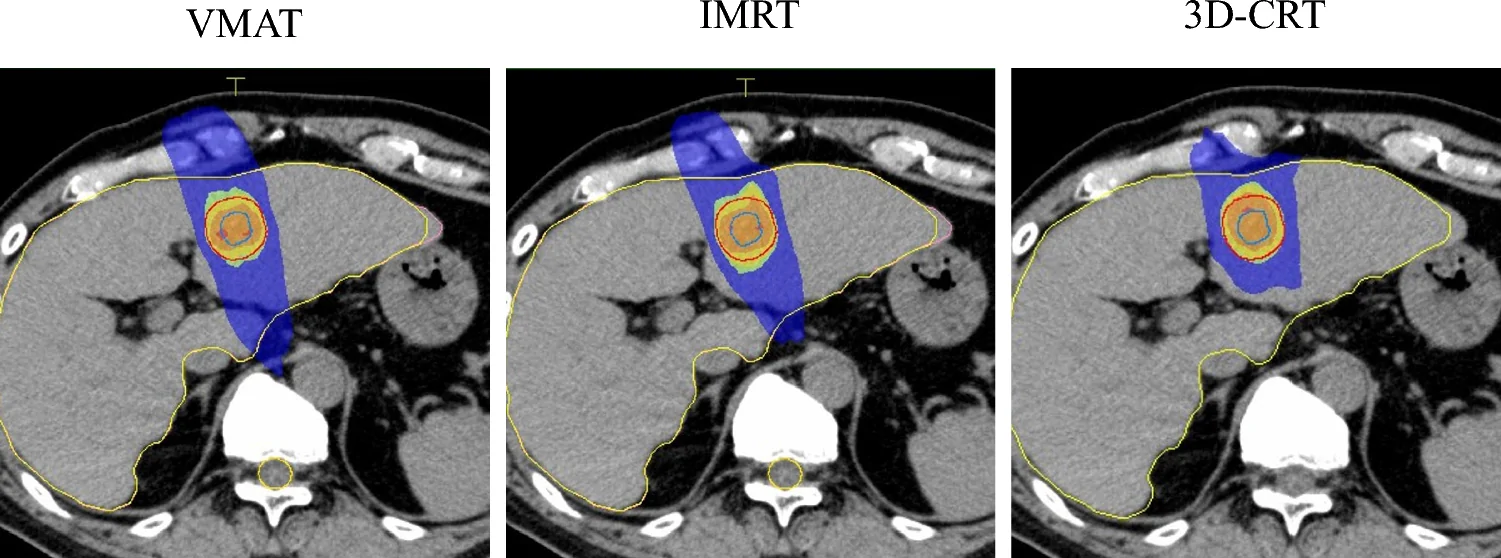
Comparison of dose distributions among treatment techniques, showing representative dose distributions for three-dimensional conformal radiotherapy (3D-CRT), intensity-modulated radiotherapy (IMRT), and volumetric modulated arc therapy (VMAT) generated using identical functional liver contours. All treatment plans achieved the prescribed target coverage (planning target volume [PTV] D95 = 40 Gy) and the maximum dose constraint (Dmax ≤ 50 Gy). Compared with 3D-CRT, IMRT and VMAT demonstrated improved dose conformity, with VMAT showing the most effective avoidance of functional liver regions while maintaining adequate PTV coverage

Representative dose distributions for the VMAT, IMRT, and 3D-CRT plans generated using identical functional liver contours are shown in Fig. 3. All treatment plans achieved the prescribed target coverage (PTV D95 = 40 Gy) and satisfied the maximum dose constraint (Dmax ≤ 50 Gy). Although adequate PTV coverage was achieved across all techniques, distinct differences in the dose distribution to the functional liver regions were visually observed among the three planning methods.

Specifically, VMAT and IMRT demonstrated similar dose distributions characterized by elongated elliptical high-dose regions. By contrast, 3D-CRT exhibited a more rounded elliptical dose distribution.

### Functional liver DVH comparison

**Fig. 4:**
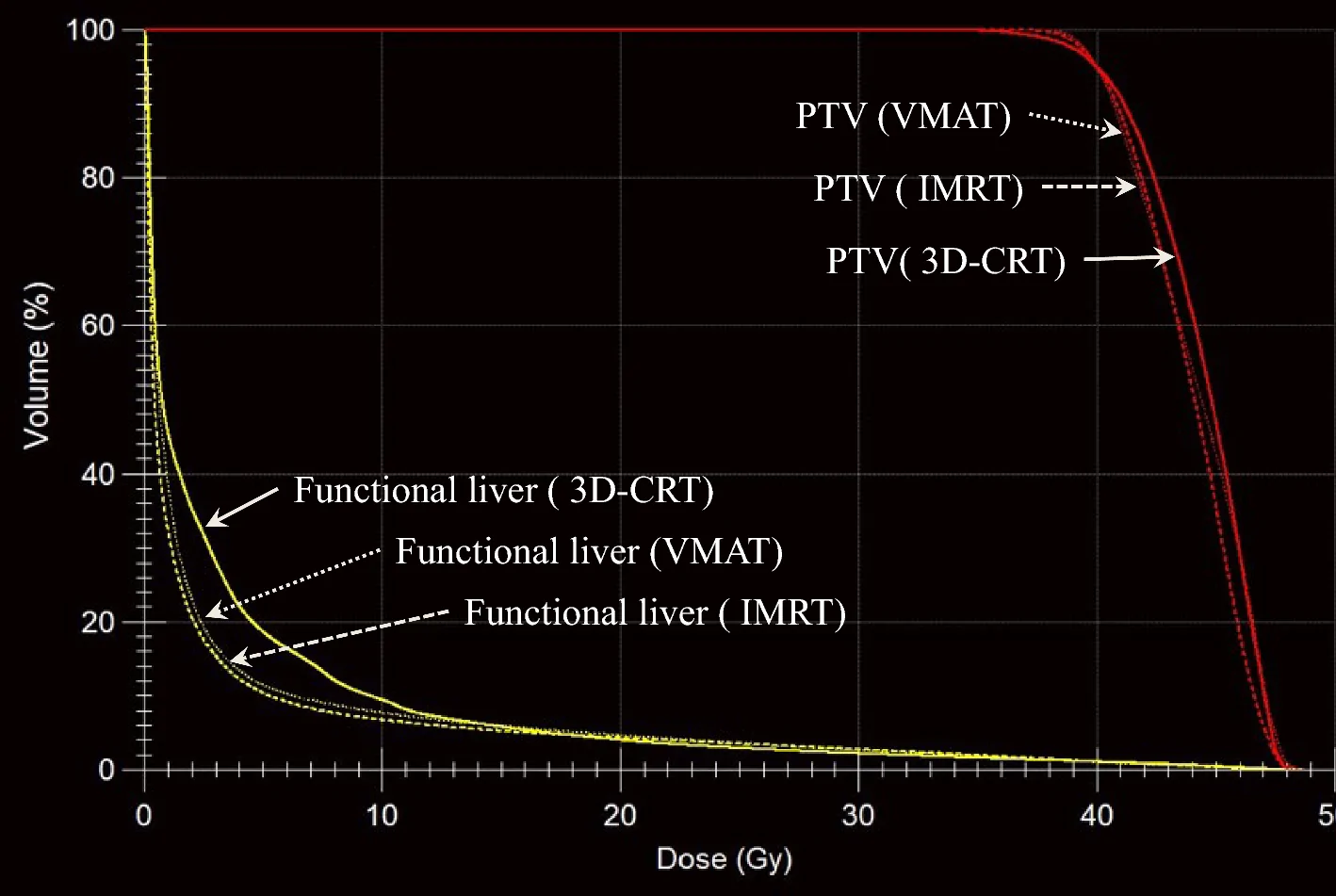
Representative dose–volume histograms (DVHs) comparing functional liver dose distributions among three-dimensional conformal radiotherapy (3D-CRT), intensity-modulated radiotherapy (IMRT), and volumetric modulated arc therapy (VMAT) for a representative patient, showing comparable planning target volume (PTV) coverage across all three techniques. In the low-dose region, VMAT and IMRT demonstrated greater reduction of functional liver irradiation compared with 3D-CRT, with VMAT and IMRT showing a similar degree of functional liver sparing

Figure 4 shows the functional liver DVHs for a representative patient comparing VMAT, IMRT, and 3D-CRT. Comparable PTV coverage was achieved using all three techniques. In the low-dose region, both VMAT and IMRT demonstrated greater and comparable reductions in functional liver irradiation than 3D-CRT. In contrast, at higher dose levels, differences among the three techniques were minimal, with little distinction observed between VMAT, IMRT, and 3D-CRT. All treatment plans satisfied the prescribed PTV dose constraints (D95 = 40 Gy and Dmax ≤ 50 Gy), resulting in comparable conformity and homogeneity indices among VMAT, IMRT, and 3D-CRT. Target coverage and conformity parameters are summarized in Table 3. All planning techniques achieved clinically acceptable target coverage and conformity.

**Table 3:** Comparison of target coverage and conformity parameters among VMAT, IMRT, and 3D-CRT plans

Parameter	VMAT	IMRT	3D-CRT	p-value
D98 (Gy)	39.3 ± 0.1	39.2 ± 0.2	38.2 ± 0.5	0.009*
D2 (Gy)	47.8 ± 0.5	47.7 ± 0.3	49.0 ± 0.9	0.042*
CI	1.1 ± 0.3	1.1 ± 0.2	1.1 ± 0.1	0.115

The table summarizes the mean values and standard deviations of PTV D98, D2, and conformity index (CI) for the VMAT, IMRT, and 3D-CRT plans. Statistical comparisons among the three planning techniques were performed using the Friedman test

*: p < 0.05

### Quantitative DVH analysis and statistical comparison

**Fig. 5:**
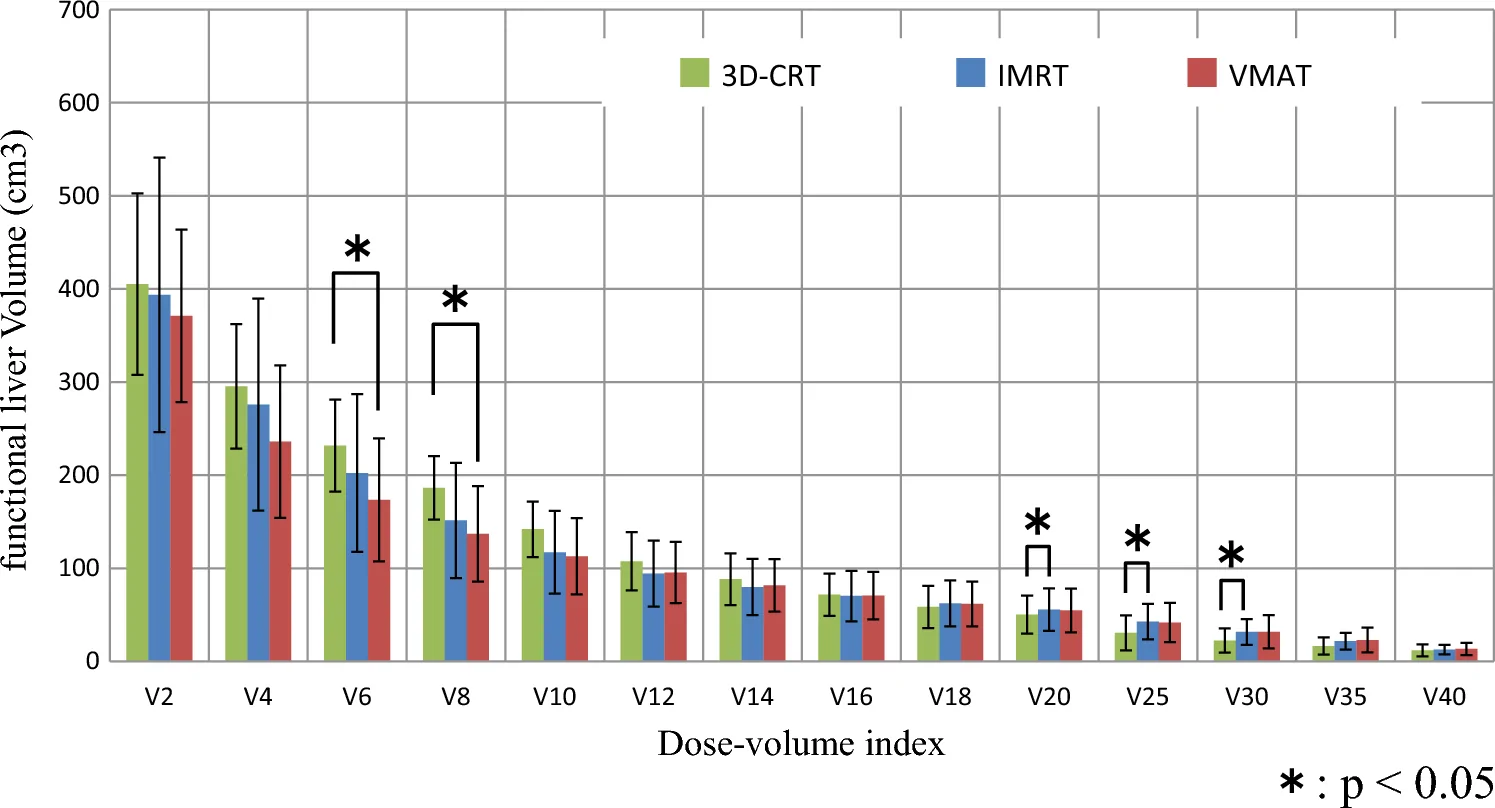
Quantitative comparison of functional liver dose–volume histogram (DVH) parameters (V2–V40) among VMAT, IMRT, and 3D-CRT across all patients. VMAT showed significantly lower functional liver volumes than 3D-CRT in the low-dose region (V6 and V8), whereas 3D-CRT demonstrated lower functional liver volumes than IMRT at higher dose levels (V20, V25, and V30), indicating dose-range–dependent differences in functional liver sparing among the three planning techniques

Quantitative comparisons of functional liver DVH parameters across all patients are illustrated in Fig. 5, and the results of statistical analyses using the Wilcoxon signed-rank test are summarized in Table 4.

**Table 4:** Wilcoxon signed-rank test results for functional liver DVH parameters

DVH parameter	VMAT vs IMRT (p)	VMAT vs 3D-CRT (p)	IMRT vs 3D-CRT (p)
V2	0.563	0.313	1.000
V4	0.063	0.063	0.563
V6	0.063	0.031*	0.156
V8	0.063	0.031*	0.094
V10	0.219	0.063	0.156
V15	0.156	0.063	0.156
V20	0.688	0.313	0.031*
V25	0.844	0.313	0.031*
V30	0.438	0.156	0.031*
V35	0.438	0.313	0.094
V40	1.000	1.000	0.563

Results of Wilcoxon signed-rank tests for functional liver dose–volume histogram (DVH) parameters (V2–V40), showing pairwise comparisons among volumetric modulated arc therapy (VMAT), intensity-modulated radiotherapy (IMRT), and three-dimensional conformal radiotherapy (3D-CRT), with asterisks indicating statistically significant differences

*: p < 0.05

In the low-dose region, VMAT resulted in significantly lower functional liver volumes than 3D-CRT at V6 and V8 (both P = 0.031). IMRT showed a similar trend toward reduced low-dose functional liver irradiation compared with 3D-CRT, although statistical significance was not consistently observed.

At higher doses, 3D-CRT demonstrated significantly lower functional liver volumes than IMRT at V20, V25, and V30 (all P = 0.031). VMAT and IMRT showed comparable functional liver volumes in the high-dose region with no significant differences between the two techniques.

Overall, VMAT provided the most favorable sparing of the functional liver in the low- to intermediate-dose regions. In contrast, 3D-CRT resulted in slightly lower functional liver exposure in the high-dose range. These results suggest that the dosimetric characteristics of each planning technique vary depending on the dose range considered, with modest technique-dependent differences observed across the low- and high-dose regions.

**Fig. 6:**
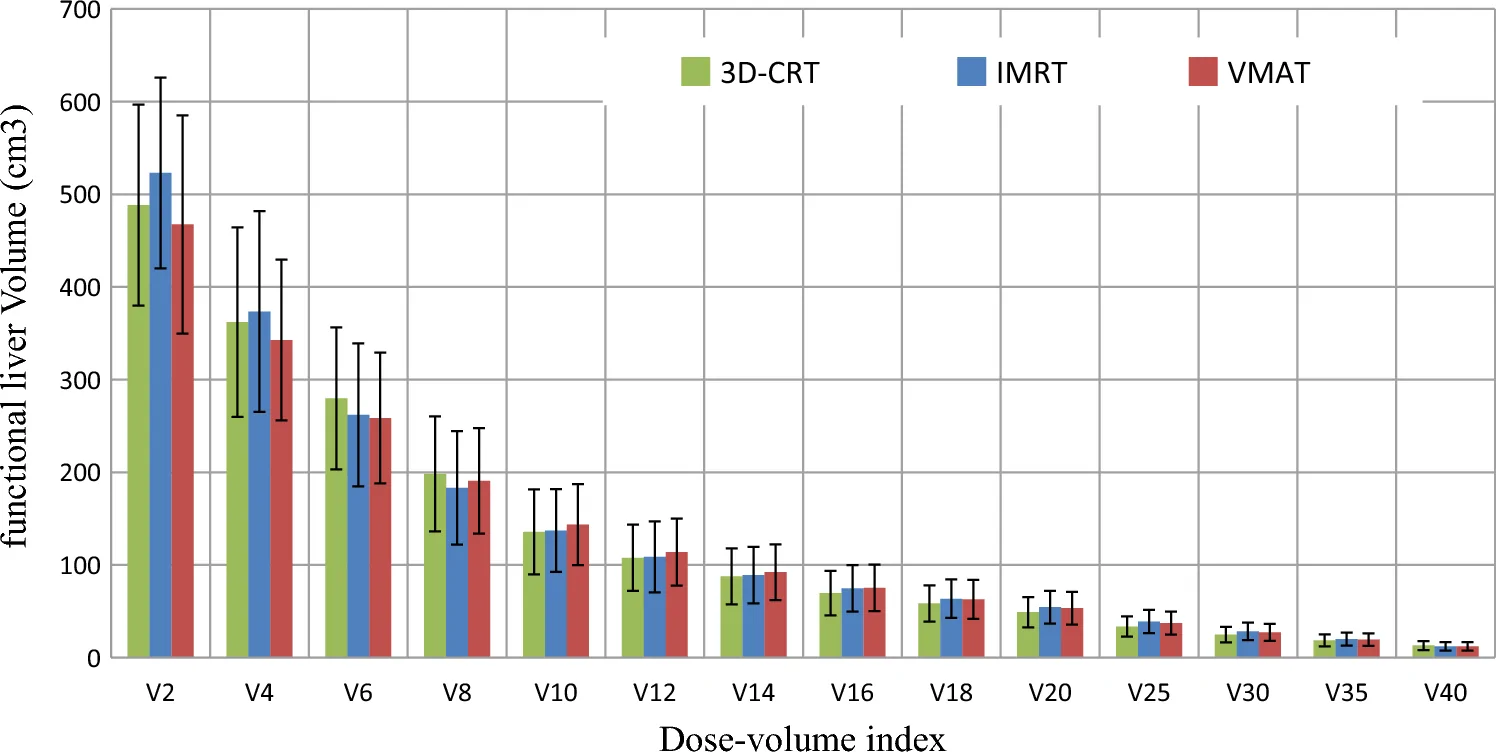
Dose–volume histogram (DVH) comparison of 3D-CRT, IMRT, and VMAT plans generated without functional liver constraints. In the absence of functional liver optimization, larger functional liver volumes were irradiated across all techniques, and no significant differences in dose–volume indices were observed among the planning methods

Figure 6 shows additional DVH analyses without functional liver constraints. Without functional liver constraints, the irradiated functional liver volume increased across all planning techniques, indicating that a larger volume of functioning liver tissue received a radiation dose. Under these conditions, no significant differences in dose-volume parameters were observed among 3D-CRT, IMRT, and VMAT. These results imply that the discrepancies observed in functional image-guided plans are primarily due to the inclusion of functional liver constraints in the treatment planning process.

## Discussion

In this study, we performed a dosimetric comparison of three radiotherapy delivery techniques—VMAT, IMRT, and 3D-CRT—using ^99m^Tc-GSA–derived functional liver information for treatment planning in patients with recurrent hepatocellular carcinoma. Our results demonstrate that the choice of delivery technique substantially influences dose distribution to functionally preserved liver regions when functional guidance is incorporated into radiotherapy planning.

Quantitative DVH analysis revealed technique-dependent differences in functional liver irradiation across dose ranges. VMAT achieved significantly lower functional liver volumes than 3D-CRT in the low-dose region, particularly at V6 and V8, whereas 3D-CRT showed significantly lower functional liver volumes than IMRT in the high-dose range at V20, V25, and V30. IMRT exhibited dose distribution characteristics that were intermediate between those of VMAT and 3D-CRT in the evaluated dose ranges. These observations suggest that dosimetric characteristics may vary depending on the dose range, rather than one technique being uniformly superior. The comparable conformity and homogeneity indices observed across techniques indicate that functional liver–sparing optimization was achieved without compromising standard target dose quality.

From a planning perspective, the relatively higher functional liver doses observed in the high-dose region for VMAT and IMRT can be explained by the characteristics of inverse planning optimization. In function-guided planning, dose reduction in functional liver regions is often achieved by preferentially routing beams through functionally impaired liver areas and assigning greater optimization weights to these beam paths. Consequently, even with multiangle or arc-based delivery, higher doses may be concentrated along specific beam trajectories, leading to a relative increase in functional liver dose in the high-dose region.

In inverse planning, these dose-dependent characteristics are strongly influenced by the definition and weighting of the dose and cost functions. In this study, functional liver–sparing objectives were applied at multiple doses (10, 20, 30, and 40 Gy). These constraints had a greater impact in the low-dose region, where the irradiated volumes were relatively large, whereas their influence was less pronounced in the high-dose region, where functional liver volumes were inherently smaller. This optimization behavior likely contributed to the marked low-dose functional liver sparing observed with VMAT and IMRT, while differences among techniques became less evident or were slightly attenuated at higher dose levels.

In conventional radiotherapy planning, liver dose constraints are generally defined under the assumption of homogeneous organ function, and treatment plans are optimized to reduce the overall liver dose. In re-irradiation settings, cumulative dose assessment is clinically important for evaluating the risk of liver toxicity [21]; however, accurate dose accumulation is challenging due to uncertainties associated with anatomical deformation and respiratory motion during the treatment course [22, 23].

In the present study, all plans were generated in compliance with guideline-based dose constraints, while functional liver information derived from ^99m^Tc-GSA SPECT was incorporated to preferentially spare regions of preserved hepatic function. Instead of relying on direct dose summation, this function-based approach enables selective dose reduction to highly functional liver regions rather than uniform dose reduction across the entire liver. This strategy reflects a shift toward individualized treatment planning based on spatial heterogeneity of liver function, which has been increasingly recognized as an important concept in recent studies [24].

Our findings are consistent with previous studies, demonstrating the feasibility and dosimetric benefits of incorporating ^99m^Tc-GSA–based functional liver imaging into radiotherapy planning. Prior reports have shown that function-guided planning can reduce irradiation of highly functional liver regions without compromising target coverage, particularly when advanced modulation techniques are employed [15–19]. Systematic reviews of functional liver imaging for radiotherapy planning have also concluded that SPECT-based approaches can improve function-weighted dosimetric parameters, although evidence linking these improvements to clinical outcomes remains limited [13]. In particular, previous DVF-based analyses using ^99m^Tc-GSA SPECT have demonstrated a dose-dependent decline in regional liver function following radiotherapy [17]. In the present study, as illustrated in Fig. 1b, reduced tracer uptake was observed in regions corresponding to the previously irradiated 40 – 44 Gy dose area, resulting in spatially heterogeneous liver function distribution. These findings are consistent with the dose–function relationship reported in previous studies.

The clinical relevance of preferentially sparing functional liver tissue is particularly important in patients with recurrent hepatocellular carcinoma undergoing re-irradiation, in whom hepatic functional reserve is often limited. Radiation-induced liver injury is influenced not only by high-dose exposure but also by the volume of functional liver receiving low- to intermediate-dose irradiation, as demonstrated in previous dose–volumetric analyses of radiation-induced liver disease following SBRT [25]. In the present study, although 3D-CRT occasionally demonstrated slightly lower functional liver exposure in the intermediate- to high-dose ranges represented by V20–V30, VMAT achieved a significant reduction in functional liver irradiation, predominantly in the low-dose ranges represented by V6 and V8 (Fig. 5). These reductions may be clinically meaningful because limiting low- and intermediate-dose exposure to functional liver tissue has been suggested to contribute to the preservation of overall hepatic function, particularly in re-irradiation settings [25].

With regard to dose constraints in the re-irradiation setting, cumulative dose summation from the initial course of radiotherapy was not explicitly performed in this study. The initial treatment consisted of stereotactic body radiotherapy, in which 40 – 44 Gy in four fractions was delivered to a limited target volume with a steep dose gradient, resulting in minimal dose exposure to the surrounding liver parenchyma. Areas corresponding to the previously irradiated 40–44 Gy dose region were consistently visualized as functional defects on ^99m^Tc-GSA SPECT, whereas adjacent liver regions demonstrated preserved tracer uptake, indicating retained hepatic function. Because conventional DVH-based evaluation assumes homogeneous organ function, regions with preserved GSA uptake were regarded as functional normal liver, even if they had been exposed to low doses during prior irradiation. Accordingly, treatment planning in the present study prioritized functional liver information derived from GSA imaging rather than cumulative physical dose constraints, with dose limitations applied specifically to regions identified as functionally preserved. This approach reflects the central concept of functional image–guided radiotherapy, in which current organ function, rather than historical dose distribution alone, is used to guide organ-at-risk sparing in re-irradiation scenarios.

This study has several limitations. First, this was a retrospective analysis with a small sample size and patient-specific variability; therefore, the statistical analyses were not intended to establish definitive conclusions, but rather to support the observed trends. Second, the analysis was purely dosimetric and did not include longitudinal clinical outcomes, such as post-treatment liver function, changes in Child–Pugh score, or the incidence of radiation-induced liver disease. Third, functional liver assessment was based on stand-alone SPECT imaging without CT-based attenuation correction, and image fusion relied on rigid registration. Although functional liver visualization and contouring were performed carefully by experienced staff, these factors may introduce uncertainty. Additionally, the degree of functional liver sparing achievable with inverse-planned techniques is highly dependent on the selection and weighting of dose constraints within the optimization process. Differences in cost function design, linear accelerator performance, and treatment planning system algorithms may also influence the balance between low- and high-dose functional liver irradiation.

Overall, the present findings demonstrate that the dosimetric characteristics of functional liver irradiation depend on the dose range rather than favoring a single treatment technique uniformly. These results underscore the importance of selecting radiotherapy techniques based on dosimetric objectives for functional liver preservation. They also support the potential role of advanced modulation techniques in optimizing treatment planning for recurrent hepatocellular carcinoma.

## Conclusion

In conclusion, this dosimetric comparison incorporating ^99m^Tc-GSA guided functional liver information indicates that inverse-planned techniques, such as VMAT and IMRT, preferentially emphasize regions of reduced hepatic function during optimization, resulting in elongated, elliptical dose distributions and superior sparing of functional liver tissue in the low-dose region. In the high-dose region represented by V35 and V40, inter-technique differences were small and did not reach statistical significance. Although 3D-CRT demonstrated significantly lower functional liver exposure in the intermediate-dose ranges represented by V20–V30, the absolute differences among techniques were similarly modest. These findings suggest that advanced modulation techniques are particularly effective in reducing low-dose functional liver irradiation (V6 and V8), whereas conventional 3D-CRT may achieve slightly lower functional liver exposure in the intermediate-dose ranges (V20–V30). Overall, ^99m^Tc-GSA SPECT–guided planning provided valuable functional information for tailoring irradiation strategies and optimizing functional liver preservation during radiotherapy for recurrent hepatocellular carcinoma.

## Data Availability

The datasets generated and/or analyzed during the current study are not publicly available. due to ethical and privacy restrictions related to patient data, but are available from the. corresponding author upon reasonable request.

## Declarations

### Prior presentation

Part of this work was presented in preliminary form at ESTRO Meets Asia 2019. The manuscript has been substantially expanded and revised for publication in your journal.
